# Determinants of muscle carnosine content

**DOI:** 10.1007/s00726-012-1233-y

**Published:** 2012-02-11

**Authors:** R. C. Harris, J. A. Wise, K. A. Price, H. J. Kim, C. K. Kim, C. Sale

**Affiliations:** 1 Junipa Ltd, Newmarket, Suffolk, CB8 8HD UK; 2 Natural Alternatives International, San Marcos, CA USA; 3 Korea National Sport University, 88-15 Oryun-dong, Songpa-gu, Seoul, Republic of Korea; 4 Nottingham Trent University, Nottingham, NG11 8NS UK

**Keywords:** Muscle carnosine, β-alanine, Species, Diet, Exercise and training, Age and gender

## Abstract

The main determinant of muscle carnosine (M-Carn) content is undoubtedly species, with, for example, aerobically trained female vegetarian athletes [with *circa* 13 mmol/kg dry muscle (dm)] having just 1/10th of that found in trained thoroughbred horses. Muscle fibre type is another key determinant, as type II fibres have a higher M-Carn or muscle histidine containing dipeptide (M-HCD) content than type I fibres. In vegetarians, M-Carn is limited by hepatic synthesis of β-alanine, whereas in omnivores this is augmented by the hydrolysis of dietary supplied HCD’s resulting in muscle levels two or more times higher. β-alanine supplementation will increase M-Carn. The same increase in M-Carn occurs with administration of an equal molar quantity of carnosine as an alternative source of β-alanine. Following the cessation of supplementation, M-Carn returns to pre-supplementation levels, with an estimated t_1/2_ of 5–9 weeks. Higher than normal M-Carn contents have been noted in some chronically weight-trained subjects, but it is unclear if this is due to the training *per se*, or secondary to changes in muscle fibre composition, an increase in β-alanine intake or even anabolic steroid use. There is no measureable loss of M-Carn with acute exercise, although exercise-induced muscle damage may result in raised plasma concentrations in equines. Animal studies indicate effects of gender and age, but human studies lack sufficient control of the effects of diet and changes in muscle fibre composition.

## Introduction

Carnosine (β-alanyl-l-histidine) is a cytoplasmic dipeptide found in high concentrations in the skeletal muscle of both vertebrates and non-vertebrates, as well as in the central nervous system. It was first isolated by Gulewitsch and Amiradzhibi (1900) and was subsequently classified as a histidine containing dipeptide (HCD) by Krimberg (1906, 1908), who demonstrated the hydrolysis of carnosine to its constituent amino acids (β-alanine and histidine). It is now known that muscle carnosine (M-Carn) is synthesised in situ (catalysed by carnosine synthase) and is limited by the availability of β-alanine. Compared with other small molecular weight compounds occurring in the millimolar range in muscle, the muscle histidine containing dipeptides (M-HCD), of which M-Carn is one, are unique in the wide range they express across species. The M-HCD’s provide a means to accumulate high, stable concentrations of histidine in muscle fibres, the imidazole ring of which is effective in buffering H^+^ over the exercise intracellular pH (pHi) transit range. M-HCD’s are the only means available to radically change the intracellular buffering capacity. Without the 6–10 times higher content of M-Carn in equine *m. gluteus medius*, compared to human *m. vastus lateralis*, pHi would be close to 5 at the end of racing, although in practice muscle contraction would most probably have ceased long before this point was reached. A high M-HCD content, and thus high-intracellular buffering capacity, would clearly be an advantage to both prey and predator where sustained activity (running, flight or diving) is important. In addition to these benefits, high M-HCD contents have been shown in several species involved in athletic competition, such as horses, greyhounds, camels and, indeed, humans (Harris et al. 1990; Dunnett and Harris 1997). Other physiological roles, in addition to intracellular pH buffering, have been suggested for M-Carn (for a brief summary see Sale et al. 2010), with the result that there may be further benefits in increasing the M-Carn content. This review will provide an overview of the major determinants of M-Carn concentrations, including species, muscle fibre type, diet, supplementation, exercise and training, gender and age.

### Species

Although beyond the scope of the individual to influence in any way, the major determinant of M-Carn, or the sum of M-HCD’s, when anserine (1-methyl carnosine) and balenine (3-methyl carnosine) are also considered, is species. For human and equine muscle, where carnosine is the sole M-HCD in both, there is typically a sixfold range in content between *m. vastus lateralis* in humans and *m. gluteus medius* in equines (Marlin et al. 1989; Harris et al. 1990, 2006; Dunnett and Harris 1997), although this is closer to 10 when the M-Carn of aerobically trained vegetarian human athletes [~13 mmol/kg dry muscle (dm)] is used as the contrast (Harris et al. 2007; Jones 2011). The range is still greater when the M-HCD content of all species is considered, approaching 40 when mouse (HCD: 5–10 mmol/dm) is compared to little piked whale (HCD: ~350 mmol/kg dm) (Abe 2000). In this respect, M-Carn, or in a more general sense M-HCD, is unique amongst the small molecular weight compounds found in the millimolar range in muscle.

The M-HCD’s provide a means to accumulate high, stable concentrations of histidine in muscle fibres, the imidazole ring of which is effective in buffering H^+^ over the pHi transit range starting at around pHi 7.1 in resting muscle and falling to pHi 6 following intense exercise (Pan et al. 1991). Changing the M-HCD content was possibly the only means available to radically change the intracellular buffering capacity in the evolution of different species, enabling the matching of ecological demands with muscle functional capacity. It can be calculated from the Henderson-Hasselbalch equation that without the 6–10 times higher content of M-Carn in equine *m. gluteus medius*, compared to human *m. vastus lateralis*, pHi would be closer to 5 at the end of racing. The calculation is made assuming a non-carnosine muscle buffering capacity of approximately 130 mmol H^+^/kg dm/pH unit change (Harris et al. 1990). A high M-HCD content, and thus high-intracellular buffering capacity, would clearly be an advantage to both prey and predator where sustained activity (running, flight or diving) is vital for survival. However, this raises the question why evolution did not ensure that all animals have M-HCD contents closer to 350 mmol/kg dm (the high level reported in little piked whale), as a ‘default level’ since most, at some point in their history, have been either prey or predator. Even in the course of recent human history, characterised as it has been by millennia of physical conflict, even a modest increase in M-Carn would have conferred advantage to an individual warrior or even a tribe. Equally, it may be questioned, what enables muscle cells of some species to accumulate and maintain M-HCD contents of 200–400 mmol/kg dm. Is this due to substrate availability for HCD synthesis or to factors preventing the leakage/destruction of HCD’s? Furthermore, would such a high content impose restrictions in other areas of metabolism? Such high levels would certainly contribute to the osmolarity of the sarcoplasm and in thoroughbred equine type II muscle fibres where M-Carn may approach 200 mmol/kg dm this is associated with a marked reduction in taurine to a level where it can be barely detected (Dunnett and Harris 1995). It was suggested that the reduction in taurine was possibly to counterbalance the osmotic effect of the high levels of M-Carn. However, given the performance capabilities of thoroughbred equines the virtual absence of taurine in type II fibres does not appear to hold any disadvantages to muscle function.

The wide variability in M-Carn or M-HCD between species parallels the periodical demand for anaerobic exercise, or tolerance to imposed hypoxic conditions (Abe 2000). For instance, in land-based mammals, higher levels are found in those species, (e.g., canines), where sustained fast running is important for hunting, and also in those species where running is important to escape, (e.g., horses and deer) (Abe 2000; Harris et al. 1990). Amongst the mammals, however, highest levels have been recorded in cetaceans (Suyama et al. 1977), where diving will impose prolonged periods of hypoxia. The same wide diversity, again linked to the periodical demand for anaerobic exercise or exposure to prolonged periods of hypoxia, is shown in other animal species. Amongst the avians, high levels of M-HCD (200–250 mmol/kg dm) are found in glycolytic pectoral muscle of pheasants, chickens and turkeys (Abe 2000; Jones 2011), where rapid beating of wings for short periods enables short periods of flight for escape. In contrast, endurance flyers such as geese and pigeon have much lower M-HCD contents (Crush 1970) consistent with the more oxidative nature of flight muscle in these species. However, the numbers of animals examined so far is still very limited and lacks key species, such as the painted turtle, which can hibernate for 3–4 months without oxygen, resulting in circulating lactate levels of 150 mmol/L (Jackson 2002).

### Muscle fibres

In those species examined so far, type II fibres have a higher M-Carn or M-HCD than type I. In equines the ratio in M-Carn between types I and II muscle fibres from *m. gluteus medius* is around five (Dunnett and Harris 1995, 1997). In humans, the type I:II ratio in fibres from *m. vastus lateralis* is 1.3–2 (Harris et al. 1998; Hill et al. 2007; Tallon et al. 2007; Kendrick et al. 2009) contributing to a small variation in M-Carn between different muscle groups. Thus, the difference in M-Carn between *m. gastrocnemius* and *m. soleus* determined by ^1^H-MRS in humans (Derave et al. 2007) is most likely a manifestation of the difference in the fibre composition between these two muscles, whilst the types I and II M-Carn contents of either may be comparatively stable. Fibre variation in M-Carn or M-HCD inevitably imposes limitations on the usefulness of measurements made at the whole muscle level where the effects of training, disuse, ageing and disease are being investigated and where changes in fibre composition may have occurred.

### Diet

M-Carn is synthesised in situ and is dependent upon the supply of β-alanine (Harris et al. 2006). In vegetarians, M-Carn is limited by hepatic synthesis of β-alanine from uracil degradation resulting in comparatively low contents of the order of 13 mmol/kg dm as measured in muscle biopsies of *m. vastus lateralis* by HPLC from a subject group mostly comprising aerobically trained female UK athletes (Harris et al. 2007; Jones 2011). Similarly, a reduced level of M-Carn in vegetarians was reported by Everaert et al. (2011) with reductions of 17–26% seen in *m. soleus*, *m. gastrocnemius* and *m. tibialis anterior*, compared with Belgian subjects eating a mixed diet. According to these authors, there was no difference in M-Carn between omnivores with a high or low ingestion of β-alanine. However, assessments of this type are difficult since they are made within a relatively uniform population and with a relatively small range in dietary β-alanine intake, calculated from meat ingestion. In addition, cooking procedures may result in 100% loss of content. It is clear from studies with β-alanine supplementation that M-Carn is highly responsive to its availability in the diet, possibly to a greater extent than any other component of metabolism in muscle, including creatine.

In omnivores, de novo hepatic β-alanine synthesis may be augmented by the hydrolysis of dietary supplied HCD’s from muscle meat resulting in levels two or more times higher than seen in vegetarians (Harris et al. 2007). Hydrolysis, in humans, of dietary supplied HCD from, for instance, the ingestion of chicken broth, has been shown to supply close to the theoretical level of the amount of β-alanine present in the bound HCD form, as evidenced by comparison of the area-under-the-plasma-concentration curve to when the same molar dose was administered in the free form (Harris et al. 2006).

### β-Alanine supplementation

The administration of free β-alanine in solution is associated with a rapid increase in the plasma concentration peaking after approximately 30–45 min irrespective of whether the dose administered was 10, 20 or 40 mg/kg body weight (Harris et al. 2006). However, above a dose of 10 mg/kg bwt subjects complained of increasing symptoms of paraesthesia, a neuropathic pain affecting areas of the face, neck, shoulders, chest and buttocks, in approximately that order. However, whilst 40 mg/kg bwt caused intense paraesthesia in all subjects tested, the same dose of β-alanine administered in the form of HCD as a chicken broth did not evoke symptoms of paraesthesia, possibly due to the altered pharmacokinetic profile, which was characterised by a delayed peak time and lower peak concentration (Harris et al. 2006).

Supplementation of the diet with β-alanine over a period of weeks will similarly increase M-Carn. In the first of a series of studies where multiple doses of 800 mg β-alanine were given per day over 4 weeks in hard gelatine capsules, the mean increase in M-Carn was 60%. When extended to 10 weeks, the increase was 80% with absolute values now close to 40 mmol/kg dm (Hill et al. 2007). A maximal single dose of 800 mg, on average 10 mg/kg bwt, was used in these studies to avoid the symptoms of paraesthesia, and is equivalent to the amount of β-alanine available from the ingestion of 150 g of chicken breast meat, assuming hydrolysis of the HCDs present. A maximum of eight such doses was given in a single day without any negative effects on subjective feelings of paraesthesia, clinical chemistry or ECG. Supplementation with carnosine (Harris et al. 2006) resulted in the same increase in M-Carn over 4 weeks of supplementation as an isomolar dose of β-alanine, showing no additional effect of the histidine also released on hydrolysis.

The absolute increase in M-Carn with 10 weeks β-alanine supplementation was the same in both type I and type II muscle fibres, despite these starting with different initial contents (Hill et al. 2007). In this study, 10 weeks β-alanine supplementation resulted in an increase in M-Carn of 16.5 mmol/kg dm in type I muscle fibres from an initial M-Carn content of 17.8 mmol/kg dm, and 17.0 mmol/kg dm in type II muscle fibres from an initial content of 29.6 mmol/kg dm. This has subsequently been confirmed in a further study where β-alanine was supplemented for 4 weeks (Kendrick et al. 2009). These results would seem to suggest that fibre-specific differences in the rates of carnosine synthesis cannot account for the differences in types I and II M-Carn contents.

With curtailment of supplementation, M-Carn returns to the pre-supplementation level in *m. vastus lateralis* with an estimated t_1/2_ of ~9 weeks (Harris et al. 2008; Stellingwerff et al. 2012). Estimates of t_1/2_ following elevation of M-Carn in *m. anterior tibialis*, *m. gastrocnemius* and *m. soleus* range from 5 to 8 weeks (Baguet et al. 2009; Stellingwerff et al. 2011, 2012). No information is available on the rates of decline of M-Carn specifically in types I and II fibres; a faster rate of decline could possibly explain the lower M-Carn content observed in type I fibres. The fate of the M-Carn accumulated during supplementation is unknown, but the possibilities are a slow release from muscle fibres or destruction within the fibres due to reaction with free radicals or carbonyl groups (Hipkiss 2000; Guiotto et al. 2005).

The responsive nature of M-Carn to exogenous β-alanine supply in humans emphasises the importance of the diet in determining its content in muscle. From the association between M-Carn and the capacity to undertake high-intensity exercise (Hill et al. 2007; Baguet et al. 2010; Sale et al. 2011), it is probable that a diet rich in β-alanine would have been advantageous to individuals in societies where extreme physical endeavour was important. As a result of the wide range in meat intake (from 0 g in vegans to perhaps 500 g per day in high meat consumers), and the marked difference in the HCD content of different meats and the influence of cooking procedures (Park et al. 2005; Purchas et al. 2004; Yeum et al. 2010; Jones 2011), the β-alanine content of current diets is estimated to vary from 0 to 1,000 mg per day. The upper value relevant to the Mongol diet (Cavendish 2007) as an example of a nomadic people eating a diet composed almost exclusively of meat (lamb, goat, chicken, horse and camel). As discussed by Wallimann et al. (2011), in the context of creatine ingestion, fossil evidence indicates that diets for the major portion of hominid evolution in northern territories were predominantly composed of meat, from which we estimate a possible dietary β-alanine intake of 1,000 mg per day or more. On this basis should 1,000 mg per day be considered normal for man even today? 1,000 mg per day is within the range, if not on the low side, of carnivores in the wild even after allowance for body weight.

Supplementation of the diet with β-alanine has also been shown to increase M-Carn in the thoroughbred race horse (Dunnett and Harris 1999), a species that would not normally encounter β-alanine in its diet. In addition, the starting pre-supplementation level of M-Carn in equine muscle is already some six times higher than that in humans (Harris et al. 1990) suggesting a higher hepatic output of β-alanine in the horse, compared to humans.

### Chronic training

Higher than normal M-Carn contents have been noted in some chronically weight-trained subjects (Parkhouse et al. 1985; Tallon et al. 2005), but it is unclear if this is due to the training per se, or secondary to changes in muscle fibre composition, an increase in meat intake or anabolic steroid use. Comparison of M-Carn measurements in Canadian sprinters, rowers and marathon runners, and untrained individuals (Parkhouse et al. 1985) with the percent fast twitch (FT) muscle fibres suggests that M-Carn in FT fibres was higher in sprinters and rowers. However, whether this was an effect of chronic training, or that sprinters and rowers ate a diet with a higher meat content, and therefore higher β-alanine content, is unclear. No measurements have been made in chronically weight-trained vegetarian athletes, which would seem to provide the best and possibly only route to establishing whether training affects M-Carn in types I and II muscle fibres.

### Acute training

Despite one report of a doubling in M-Carn with 8 weeks training consisting of 28 bouts of multiple 30 s Wingate tests performed on a cycle ergometer, and with a total exercise time over the 8 weeks of 14 min (Suzuki et al. 2004), other studies using 4–16 weeks intensive sprint training (Kendrick et al. 2009; Mannion et al. 1994), 12 weeks whole body training (Kendrick et al. 2008) and 4 weeks unilateral cycle training (Kendrick 2011) failed to show any response of M-Carn to acute training. This was confirmed also at the level of types I and II muscle fibres.

Supplementation with β-alanine during 4 and 12 weeks of training resulted, as expected, in an increase in M-Carn (Kendrick et al. 2008, 2009), but the increase was the same as in the absence of training. The lack of any enhanced response to β-alanine supplementation by training was confirmed also in types I and II muscle fibres (Kendrick et al. 2009). The lack of any training effect even after 16 weeks suggests no increase occurs in the hepatic synthesis of β-alanine during this time, as would be needed to support a higher rate of M-Carn synthesis. The absence of any further increase in M-Carn synthesis with training combined with β-alanine supplementation suggests that the transport of β-alanine into fibres is unaffected by acute training, and possibly also the expression of the enzyme in muscle responsible for carnosine synthesis (carnosine synthase). More recently Baguet et al. (2011a) reported a small but non-significant increase of M-Carn in *m. soleus*, but not in *m. gastrocnemius* or *m. anterior tibialis*, with 5 weeks of training comprising 2–3 bouts per week, using ^1^H-MRS and with subjects maintained on a mixed diet. The increase in *m. soleus* was transformed into a non-significant decrease of 9% when subjects were maintained on a vegetarian diet during the period of training. The results confirm that hepatic β-alanine synthesis in subjects fed with a vegetarian diet can barely support the carnosine synthesis rate observed in omnivores and further was not increased with 5 weeks of training. In this study, carnosine synthase mRNA expression was shown to be independent of training, but decreased significantly in the vegetarian group.

### Acute excercise

In equines there is no measureable loss of M-Carn with acute exercise (Harris et al. 1991), although exercise-induced muscle damage may result in raised plasma concentrations in equines (Dunnett et al. 2002). No comparable studies have been performed in humans, which would necessitate measurements of M-Carn in freeze-dried muscle biopsies to obviate the effects of exercise induced hyperaemia and changes in muscle water content. However, it is clear that there is no progressive loss of M-Carn with chronic training of 12–16 weeks duration (Kendrick et al. 2009; Mannion et al. 1994).

### Gender

In rats, a lower level of M-HCD was observed in hind limb, fore limb and pectoral muscle of females compared to males by Peñafiel et al. (2004). Castration of male rats resulted in a 21% fall in the M-HCD content, whereas administration of testosterone propionate to female rats resulted in an almost threefold increase in M-HCD content. The differences seen in male and female rats and the effects of castration and testosterone administration are far greater than might be accounted for by shifts in muscle fibre composition, and as the diet was the same in all situations, it seems safe to conclude that in rats there is an effect of gender. However, whether this is due to changes in the expression of muscle located factors, or due to gender or testosterone induced differences in hepatic β-alanine synthesis rates is unclear.

An effect of gender is less clear in equines where only a small trend to greater M-Carn was observed in *m. gluteus medius* of 1-year-old colts compared to 1-year-old fillies (Marlin et al. 1989). No differences were seen between trained and lightly trained 2 and 3 year old male and female thoroughbreds. In humans, Mannion et al. (1992) observed an 18% reduction in M-Carn in biopsies of *m. vastus lateralis* from females compared to males, and Everaert et al. (2011) observed reductions of ~20, ~23 and ~45% in *m. soleus, m. gastrocnemius* and *m. anterior tibialis* , respectively, determined by ^1^H-MRS (recalculated from the data of Everaert et al. 2011). M-Carn was unrelated to testosterone concentrations in the circulation. Data from the same group (Baguet et al. 2011b) showed that M-Carn was elevated during puberty in boys, but not in girls. In boys, M-Carn was increased from pre-puberty to adolescence by 22.9% in *m. gastrocnemius* and 44.6% in *m. soleus* muscles. These data are thus in line with the data from murine muscle, as reported by Peñafiel et al. (2004). However, these data were performed on whole muscle and did not take into account possible changes in type II muscle fibre expression. Increases in M-Carn with puberty could thus be secondary to changes in muscle fibre expression. As yet only one study has investigated the M-Carn of types I and II muscle fibres from male and female subjects (Tallon et al. 2007). In this study, no differences were observed between male and female subjects in the age range 20–35 years in the M-Carn content of types I and II muscle fibres from *m. vastus lateralis*. Although comprising just four females and five males, the study suggested that the apparent gender difference reported by Everaert et al. (2011) may have been simply due to a higher type I:II ratio in females in the voxel sampled. It seems unlikely that measurements of M-Carn at the whole muscle level alone will be sufficient to establish the effect of gender.

Differences in diet between male and female subjects should not also be discounted in view of the responsiveness of M-Carn to even small differences in the β-alanine content of the diet. Again this could be a confounding factor in assessing whether or not there is an effect of gender. Indeed, the highest M-Carn contents observed in biopsy samples of *m. vastus lateralis* by Jones (2011) were in Australian female students who reportedly ate a diet with a high (by European standards) meat content. Confirmation of the effect of gender should be undertaken in long-term vegetarian subjects and should include measurements of types I and II muscle fibres. Measurements of hepatic β-alanine synthesis rates in males and females should also be included.

### Age

Stuerenburg and Kunze (1999) were the first to report that M-HCD declined with age in healthy rats. Derave et al. (2008) observed a decrease of 45% in M-HCD in *m. tibialis anterior* of male senescence-accelerated mice, seemingly confirming the decline in M-HCD content in murine muscle. In humans Stuerenburg and Kunze (1999) observed a negative correlation of M-Carn with age in humans with motor neuron disease. Similarly, Everaert et al. (2011) showed a trend to lower levels in *m. soleus* from healthy Belgian subjects aged 19–47 years. Data from the same group has confirmed an ageing effect on M-Carn, but suggests that this decline begins during middle age rather than old age (Baguet et al. 2011b). However, in neither study were measurements made on types I and II muscle fibres nor was there any attempt to eliminate the possibility that the decline was linked to dietary changes or reduced hepatic β-alanine synthesis. To date, only one study has attempted to investigate changes in M-Carn with age in types I and II muscle fibres (Tallon et al. 2007). Compared to inactive males aged 23 ± 5 years, older subjects (58–67 years) with osteoarthritis of the knee showed a marked reduction in M-Carn in type II fibres; whereas M-Carn in type I fibres was unchanged. To understand the nature of the changes in M-Carn with age, future studies should include measurements in type I and II muscle fibres, as well as strict control of the diet. In a study of older Korean males aged 58–67 years, and with impaired glucose tolerance, M-Carn levels were in the same range as those found in elite male Korean swimmers (Kim 2009). It was suggested that the dietary intake of β-alanine was probably sufficiently high in the elderly group as to override any trend to a lower M-Carn with age. As reported in this issue by del Favero et al. (2011), β-alanine supplementation is also effective in raising the M-Carn content in elderly subjects (65 ± 4 years).

## Conclusion and directions for future studies

Studies of M-Carn in humans are compounded by its distribution in different fibre types and its response to exogenous β-alanine supply. As a result of the low turnover rate (*sic.* washout) of M-Carn, slow accumulation of M-Carn may be possible with even modest increases in exogenous β-alanine supply. National differences in dietary habits, including meat intake, whether eaten raw, boiled with the stock removed, or roasted, are likely to affect dietary β-alanine intake. It is therefore important to note the regional, and at times ethnic, make-up of subjects being investigated, and to supply estimates of dietary β-alanine intake. This could be overcome by studying long-term vegetarian subjects (young, elderly, trained and untrained) and to supply them with a background supply over several months, of β-alanine of 0, 200, 400 and 800 mg corresponding to vegetarian, low, medium and moderately high-dietary intakes.

Differences in fibre composition may compound the effects of chronic training and ageing, along with dietary changes. In such studies it would seem mandatory to include measurements of M-Carn in separated pools of types I and II muscle fibres. Where ^1^H-MRS is used then issues relating to hydration and fat infiltration of muscle, particularly in the elderly; water shifts associated with acute exercise; changes in glycogen storage and creatine accumulation, need to be accounted for. In addition, other effects such as exercise-induced hyperaemia should be taken into account with studies of acute exercise. In short, the evaluation of those factors determining the M-Carn content of human muscle need to be undertaken with care. In addition, we should give consideration to the wide diversity in human diets, both present and in the past, noting also what may have been typical for the major time of hominid evolution (see Wallimann et al. 2011 for a similar consideration of creatine in the hominid diet).

For understanding the role of carnosine in muscle one would be advised to greatly broaden the spectrum of species already examined, including (in the future) studies of muscle from species faced by prolonged periods of hypoxia, e.g., the painted turtle, from sprinters such as the cat family, and, of muscle with greatly increased oxidative capacity, e.g., *m. pectoralis* from humming birds. Carnosine is a molecule capable of pleiotropic effects. Today we know it to be an important contributor to intracellular acid–base regulation, but whether other effects are manifest in vivo at the physiological level in human muscle is currently unclear.
